# Supplementary material for: Gut microbial trimethylamine is elevated in alcohol-associated hepatitis and contributes to ethanol-induced liver injury in mice
Source: eLife. 2022 Jan 27;11:e76554. doi: 10.7554/eLife.76554 (PMC8853661; doi:10.7554/eLife.76554)
Supplement: Figure 1—source data 1. [file elife-76554-fig1-data1.docx]

**Figure 1-source data 1: Demographic and clinical parameters for entire cohort of healthy controls and patients with AH (including DASH and NOAC biorepository enrollees).**

|  | Healthy Control (N=21) | Alcohol Hepatitis Moderate (N=112) | Alcohol Hepatitis Severe (N=152) | Total (N=285) |
| --- | --- | --- | --- | --- |
| **Race (N, %)** |  |  |  |  |
| African American | 0 (0.0%) | 6 (5.4%) | 12 (7.9%) | 18 (6.3%) |
| Asian | 1 (4.8%) | 1 (0.9%) | 0 (0.0%) | 2 (0.7%) |
| Other | 0 (0.0%) | 1 (0.9%) | 1 (0.7%) | 2 (0.7%) |
| White | 20 (95.2%) | 104 (92.9%) | 139 (91.4%) | 263 (92.3%) |
| **Gender (N, %)** |  |  |  |  |
| Female | 12 (57.1%) | 48 (42.9%) | 62 (40.8%) | 122 (42.8%) |
| **Age (years)** |  |  |  |  |
| Median | 42 | 49.5 | 48 | 49 |
| Mean (SE) | 43.09 (3.18) | 48.42 (1.06) | 46.37 (0.8) | 46.937 (0.64) |
| **Site (N, %)** |  |  |  |  |
| CCF | 21 (100%) | 28 (25.0%) | 48 (31.6%) | 97 (34.0%) |
| Louisville | 0 (0.0%) | 9 (8.0%) | 13 (8.6%) | 22 (7.7%) |
| UMMS | 0 (0.0%) | 49 (43.8%) | 35 (23.0%) | 84 (29.5%) |
| UTSW | 0 (0.0%) | 26 (23.2%) | 56 (36.8%) | 82 (28.8%) |
| **AUDIT Score**^†^  Median  Mean (SE) | ND  ND | 25  22.03 (1.61) | 25  23.03 (1.07) | 25  22.70 (0.88) |
| **Laboratory Results** |  |  |  |  |
| **Bilirubin (mg/dL)** |  |  |  |  |
| Median | ND | 3.30 | 17.35 | 11.60 |
| Mean (SE) | ND | 5.06 (0.52) | 18.87 (0.74) | 13.06 (0.64) |
| **AST (U/L)** |  |  |  |  |
| Median | ND | 78 | 110.5 | 99 |
| Mean (SE) | ND | 99.66 (7.09) | 131.13 (10.24) | 117.88 (6.70) |
| **ALT (U/L)** |  |  |  |  |
| Median | ND | 34 | 40.5 | 39 |
| Mean (SE) | ND | 45.42 (3.51) | 49.11 (3.03) | 47.56 (2.29) |
| **INR**^††^ |  |  |  |  |
| Median | ND | 1.2 | 1.8 | 1.6 |
| Mean (SE) | ND | 1.29 (0.03) | 1.912 (0.04) | 1.65 (0.03) |
| **Creatinine (mg/dL)** |  |  |  |  |
| Median | ND | 0.71 | 0.79 | 0.75 |
| Mean (SE) | ND | 0.75 (0.03) | 1.11 (0.08) | 0.96 (0.05) |
| **Albumin (g/dL)** |  |  |  |  |
| Median | ND | 3.10 | 2.60 | 2.70 |
| Mean (SE) | ND | 3.22 (0.07) | 2.64 (0.04) | 2.88 (0.04) |
| **Alkaline phosphatase (U/L)** |  |  |  |  |
| Median | ND | 130.00 | 153.50 | 146.00 |
| Mean (SE) | ND | 150.89 (8.38) | 178.543 (8.734) | 166.59 (6.19) |
| **White blood cells (10^3^/mm^3^)** |  |  |  |  |
| Median | ND | 6.20 | 9.34 | 8.14 |
| Mean (SE) | ND | 7.27 (0.37) | 11.68 (0.58) | 9.82 (0.40) |
| **Total Protein (g/dL)** |  |  |  |  |
| Median | ND | 6.70 | 6.00 | 6.30 |
| Mean (SE) | ND | 6.68 (0.11) | 5.96 (0.07) | 6.27 (0.07) |
| **Prognostic Scores** |  |  |  |  |
| **MELD Score**^§^  Median  Mean (SE) | ND  ND | 14  13.50 (0.42) | 25  25.96 (0.41) | 22  20.76 (0.48) |
| **Child-Turcotte Pugh Score**^¶^  Median  Mean (SE) | ND  ND | 8  7.81 (0.11) | 10  10.53 (0.11) | 10  9.36 (0.13) |
| **Maddrey’s Discriminant Function**^‡^  Median  Mean (SE) | ND  ND | 12.30  12.32 (1.30) | 52.64  56.80 (2.14) | 35.11  37.63 (1.94) |
| † AUDIT Questionnaire = Alcohol Use Disorders Identification Test  ^††^ INR: international normalized ratio (INR) is a calculation based on results of a Prothrombin Time  § In the Model for End-Stage Liver Disease (MELD), scores range from 6 to 40, with higher scores indicating worse prognosis.  ¶ Child-Turcotte-Pugh Score classes for cirrhosis severity: A = 5-6 points; B = 7-9 points; C = 10-15 points  ‡ Maddrey’s Discriminant function is calculated as 4.6 × (patient’s prothrombin time in seconds − lab control’s prothrombin time in seconds) + patient’s serum bilirubin level in milligrams per deciliter; a value of 32 or higher indicates severe alcoholic hepatitis that carries an adverse prognosis. | | | | |
