# Supplementary material for: Gut microbial trimethylamine is elevated in alcohol-associated hepatitis and contributes to ethanol-induced liver injury in mice
Source: eLife. 2022 Jan 27;11:e76554. doi: 10.7554/eLife.76554 (PMC8853661; doi:10.7554/eLife.76554)
Supplement: Figure 1—source data 2. [file elife-76554-fig1-data2.docx]

**Figure 1-source data 2: Demographic and clinical parameters for subset of healthy controls and patients with AH (including DASH and NOAC biorepository enrollees) included in TMA assay.**

|  | Healthy Control (N=13) | Alcohol Hepatitis Moderate (N=52) | Alcohol Hepatitis Severe (N=83) | Total (N=148) |
| --- | --- | --- | --- | --- |
| **Race (N, %)** |  |  |  |  |
| African American | 0 (0.0%) | 3 (5.8%) | 5 (6.0%) | 8 (5.4%) |
| Asian | 1 (7.7%) | 0 (0.0%) | 0 (0.0%) | 1 (0.7%) |
| Other | 0 (0.0%) | 1 (1.9%) | 0 (0.0%) | 1 (0.7%) |
| White | 12 (92.3%) | 48 (92.3%) | 78 (94.0%) | 138 (93.2%) |
| **Gender (N, %)** |  |  |  |  |
| Female | 7 (53.8%) | 24 (42.3%) | 33 (39.8%) | 62 (41.9%) |
| **Age (years)** |  |  |  |  |
| Median | 42 | 49 | 47 | 48 |
| Mean (SE) | 41.4 (4.2) | 47.46 (1.75) | 45.96 (1.04) | 46.08 (0.87) |
| **Site (N, %)** |  |  |  |  |
| CCF | 13 (100%) | 12 (23.1%) | 15 (18.1%) | 40 (27.0%) |
| Louisville | 0 (0.0%) | 0 (0.0%) | 0 (0.0%) | 0 (0.0%) |
| UMMS | 0 (0.0%) | 21 (40.4%) | 28 (33.7%) | 49 (33.1%) |
| UTSW | 0 (0.0%) | 19 (36.5%) | 40 (48.2%) | 59 (39.9%) |
| **AUDIT Score**^†^  Median  Mean (SE) | ND  ND | 23  19.86 (2.35) | 24  22.22 (1.66) | 24  21.42 (1.35) |
| **Laboratory Results** |  |  |  |  |
| **Bilirubin (mg/dL)** |  |  |  |  |
| Median | ND | 4.10 | 16 | 11.50 |
| Mean (SE) | ND | 4.49 (0.52) | 18.18 (1.01) | 12.99 (0.88) |
| **AST (U/L)** |  |  |  |  |
| Median | ND | 78 | 99 | 93 |
| Mean (SE) | ND | 99.42 (10.45) | 118.15 (8.06) | 111.00 (6.41) |
| **ALT (U/L)** |  |  |  |  |
| Median | ND | 31 | 45 | 38 |
| Mean (SE) | ND | 43.98 (5.09) | 49.23 (4.22) | 47.23 (3.25) |
| **INR**^††^ |  |  |  |  |
| Median | ND | 1.25 | 1.90 | 1.60 |
| Mean (SE) | ND | 1.34 (0.05) | 1.97 (0.06) | 1.74 (0.05) |
| **Creatinine (mg/dL)** |  |  |  |  |
| Median | ND | 0.71 | 0.81 | 0.77 |
| Mean (SE) | ND | 0.766 (0.04) | 1.04 (0.11) | 0.94 (0.07) |
| **Albumin (g/dL)** |  |  |  |  |
| Median | ND | 3.30 | 2.60 | 2.70 |
| Mean (SE) | ND | 3.26 (0.11) | 2.70 (0.06) | 2.92 (0.06) |
| **Alkaline phosphatase (U/L)** |  |  |  |  |
| Median | ND | 128.00 | 147.00 | 140.50 |
| Mean (SE) | ND | 140.37 (10.42) | 170.74 (10.24) | 159.29 (7.58) |
| **White blood cells (10^3^/mm^3^)** |  |  |  |  |
| Median | ND | 6.00 | 8.86 | 7.60 |
| Mean (SE) | ND | 7.29 (0.53) | 10.75 (0.73) | 9.51 (0.53) |
| **Total Protein (g/dL)** |  |  |  |  |
| Median | ND | 6.70 | 6.00 | 6.20 |
| Mean (SE) | ND | 6.65 (0.16) | 6.02 (0.10) | 6.25 (0.09) |
| **Prognostic Scores** |  |  |  |  |
| **MELD Score**^§^  Median  Mean (SE) | ND  ND | 16  14.08 (0.66) | 25  25.54 (0.56) | 22  21.28 (0.65) |
| **Child-Turcotte Pugh Score**^¶^  Median  Mean (SE) | ND  ND | 8  8.043 (0.26) | 10  10.41 (0.13) | 10  9.53 (0.16) |
| **Maddrey’s Discriminant Function**^‡^  Median  Mean (SE) | ND  ND | 14.42  13.50 (1.79) | 51.88  56.54 (2.88) | 36.57  40.56 (2.64) |
| † AUDIT Questionnaire = Alcohol Use Disorders Identification Test  ^††^ INR: international normalized ratio (INR) is a calculation based on results of a Prothrombin Time  § In the Model for End-Stage Liver Disease (MELD), scores range from 6 to 40, with higher scores indicating worse prognosis.  ¶ Child-Turcotte-Pugh Score classes for cirrhosis severity: A = 5-6 points; B = 7-9 points; C = 10-15 points  ‡ Maddrey’s Discriminant function is calculated as 4.6 × (patient’s prothrombin time in seconds − lab control’s prothrombin time in seconds) + patient’s serum bilirubin level in milligrams per deciliter; a value of 32 or higher indicates severe alcoholic hepatitis that carries an adverse prognosis. | | | | |
