# Supplementary figures and images for: Gut microbial trimethylamine is elevated in alcohol-associated hepatitis and contributes to ethanol-induced liver injury in mice
Source: eLife. 2022 Jan 27;11:e76554. doi: 10.7554/eLife.76554 (PMC8853661; doi:10.7554/eLife.76554)

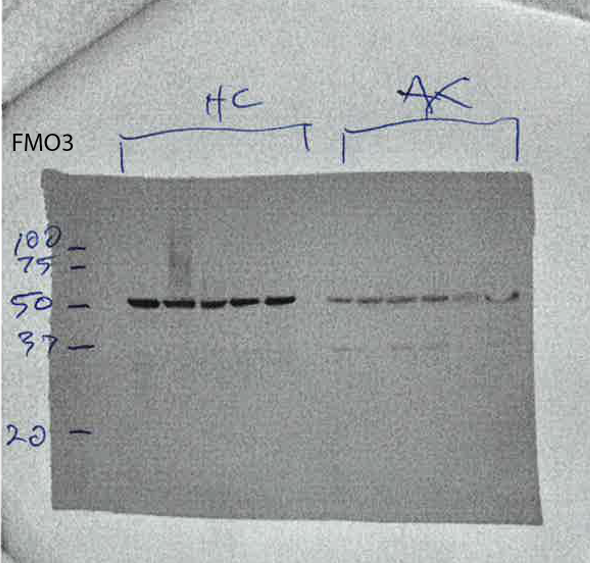

Supplement: Figure 1—source data 3. — These data are showing the entire blot. [file elife-76554-fig1-data3.zip › Figure 1-source data 3.tif]

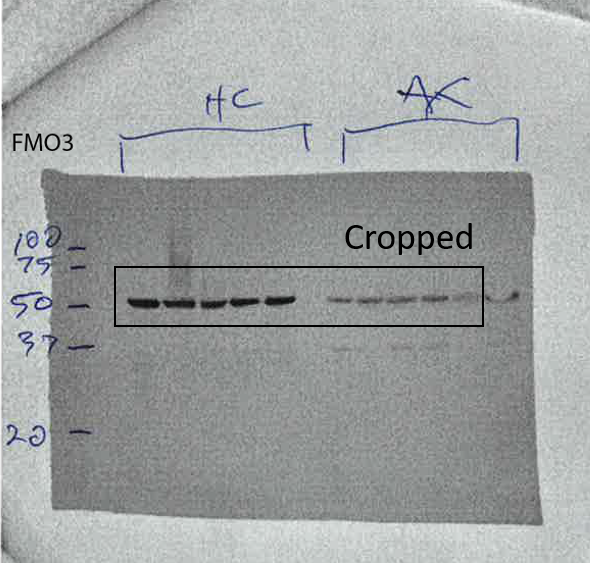

Supplement: Figure 1—source data 4. — These data are showing the cropped version of the blot. [file elife-76554-fig1-data4.zip › Figure 1-source data 4.tif]

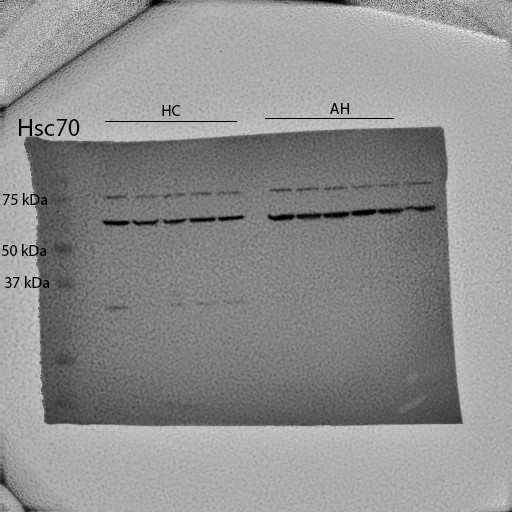

Supplement: Figure 1—source data 5. — These data are showing the entire blot. [file elife-76554-fig1-data5.zip › Figure 1-source data 5.tif]

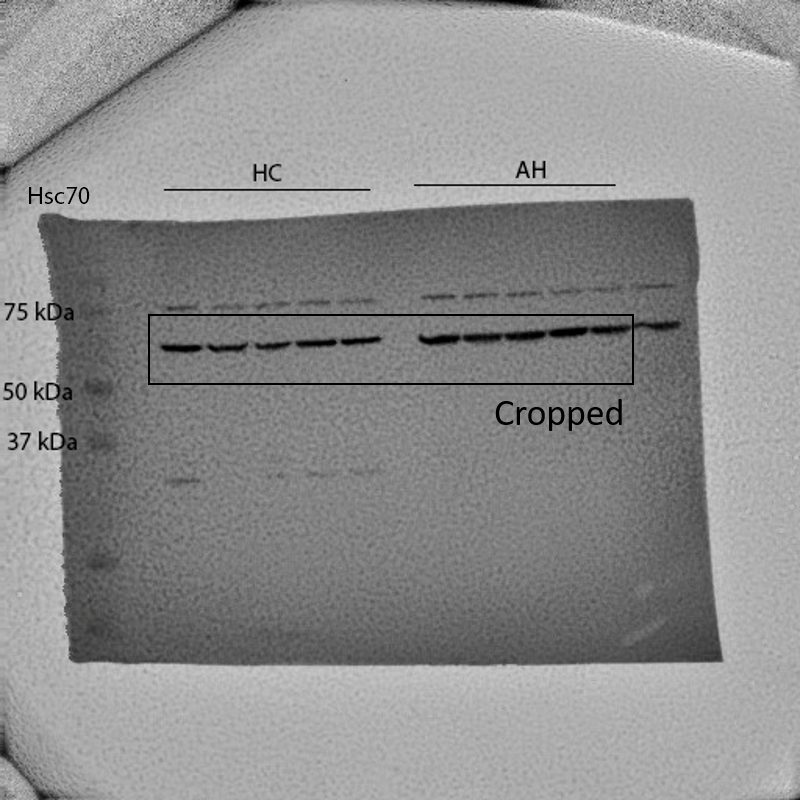

Supplement: Figure 1—source data 6. — These data are showing the cropped version of the blot. [file elife-76554-fig1-data6.zip › Figure 1-source data 6.tif]
